# Assessing kinetic fractionation in brachiopod calcite using clumped isotopes

**DOI:** 10.1038/s41598-017-17353-7

**Published:** 2018-01-11

**Authors:** David Bajnai, Jens Fiebig, Adam Tomašových, Sara Milner Garcia, Claire Rollion-Bard, Jacek Raddatz, Niklas Löffler, Cristina Primo-Ramos, Uwe Brand

**Affiliations:** 1 Institut für Geowissenschaften, J. W. Goethe-Universität, Altenhöferallee 1, 60438 Frankfurt am Main, Germany; 2 Earth Science Institute, Slovak Academy of Sciences, Dúbravská cesta 9, 84005 Bratislava, Slovakia; 3 Institut de Physique du Globe de Paris (IPGP), UMR CNRS 7154, Université Paris Diderot, 1 rue Jussieu, 75238, Paris, CEDEX 05 France; 4 Senckenberg Biodiversity and Climate Research Centre (BiK-F), Senckenberganlage 25, 60325 Frankfurt am Main, Germany; 5 Institut für Atmosphäre und Umwelt, J. W. Goethe-Universität, Altenhöferallee 1, 60438 Frankfurt am Main, Germany; 6 Department of Earth Sciences, Brock University, 1812 Sir Isaac Brock Way, L2S 3A1 St. Catharines, Ontario, Canada

## Abstract

Brachiopod shells are the most widely used geological archive for the reconstruction of the temperature and the oxygen isotope composition of Phanerozoic seawater. However, it is not conclusive whether brachiopods precipitate their shells in thermodynamic equilibrium. In this study, we investigated the potential impact of kinetic controls on the isotope composition of modern brachiopods by measuring the oxygen and clumped isotope compositions of their shells. Our results show that clumped and oxygen isotope compositions depart from thermodynamic equilibrium due to growth rate-induced kinetic effects. These departures are in line with incomplete hydration and hydroxylation of dissolved CO_2_. These findings imply that the determination of taxon-specific growth rates alongside clumped and bulk oxygen isotope analyses is essential to ensure accurate estimates of past ocean temperatures and seawater oxygen isotope compositions from brachiopods.

## Introduction

Biomineralising marine organisms serve as important geochemical archives of past climate conditions. Brachiopods constitute one group of calcifying invertebrates that have great potential for palaeoenvironmental reconstructions due to their common occurrences in Phanerozoic sediments since the Cambrian^1^. Their high abundance in Palaeozoic sediments makes them particularly valuable for deep-time seawater temperature reconstructions based on shell oxygen isotope compositions^2^. Unlike many other biogenic archives fossil and modern brachiopods can be found from tropical to polar environments and from a great range of water depths^1,3^.

A limitation of the conventional oxygen isotope palaeothermometer method is that it requires an assumption for the oxygen isotope composition of the palaeo-seawater^4^. The common assumption that the seawater δ^18^O_VSMOW_ values remained constantly between −1‰ and 0‰ during the Phanerozoic leads to relatively low apparent oxygen isotope fractionation between ancient seawater and brachiopod calcite, and hence to unrealistically high seawater temperature estimates^2^. Alternatively, it has been claimed that the progressive ^18^O depletion of brachiopod shells with age during the Phanerozoic reflects increasing post-depositional alteration or a secular decline in seawater δ^18^O values of about −6‰ compared to the modern ocean^2^. To investigate the underlying cause of presumably erroneous extremely warm Phanerozoic temperature estimates, independent constraints on past seawater temperatures and δ^18^O values are needed.

In contrast to oxygen isotope thermometry, the carbonate clumped isotope thermometer does not require an estimate for the oxygen isotope composition of the seawater, as it considers the fractionation of isotopes exclusively amongst carbonate isotopologues^5^. In thermodynamic equilibrium, the clumped isotope composition (∆_47_) of a given carbonate is solely a function of the carbonate precipitation temperature. Fossil brachiopod shells have been analysed both for both their oxygen and clumped isotope composition to independently constrain ocean temperatures and seawater δ^18^O^6–8^. However, previous investigations into the temperature dependence of the clumped isotope composition of modern brachiopod shells have reported inconsistent results. Came *et al*.^9^ reported a significantly steeper ∆_47_–temperature slope compared to the theoretical calibration^10^ and to the empirical calibration based on brachiopods and molluscs^11^. Came *et al*.^9^ exclusively investigated brachiopods, whereas the calibration of Henkes *et al*.^11^ was primarily based on molluscs. Differences in phosphoric acid digestion temperatures (90 °C *vs*. 25 °C) were suggested as a possible explanation for the discrepant ∆_47_–temperature slopes^9^. However, it remains an open question whether kinetic fractionation processes may account for the observed discrepancies in the ∆_47_–temperature slopes.

Kinetic isotope fractionations driven by diffusion, pH or incomplete oxygen isotope exchange between water and dissolved inorganic carbonate species can cause calcite to be precipitated with isotope values that are offset from those predicted for thermodynamic equilibrium^12–14^. Kinetic fractionation effects have been recognised in other important calcifying groups, including in warm and cold-water corals and in certain foraminifera species^12,14–16^. It has been postulated that brachiopods incorporate oxygen isotopes into shell calcite (secondary and tertiary layers) in equilibrium with ambient seawater, although certain parts of the shell (i.e., primary layer, uppermost part of the secondary layer, umbo and muscle scar areas) yield depleted δ^18^O values^17–21^. In these shell areas, the observed ^18^O-depletion has been linked to growth-rate-driven kinetic isotope fractionation^18,22–26^.

Here, we investigate the significance of kinetic controls (also called vital effects) on brachiopod shell ∆_47_ and δ^18^O values. We analysed the bulk and clumped isotope compositions of eighteen modern brachiopod shells at a phosphoric acid digestion temperature of 90 °C. The studied specimens represent fourteen species collected from different geographic locations and water depths that cover a substantial range of growth temperatures (Supplementary Table 1). Growth temperatures and seawater δ^18^O values for each brachiopod were independently-determined and we complemented our measurements with trace element and ion probe-based *in situ* oxygen isotope analyses.

## Results

### Trace element analyses

The magnesium concentration of the studied modern brachiopod shells was between 0.27 mol% (*Terebratalia transversa*) and 6.8 mol% (*Pajaudina atlantica*) MgCO_3_. Our results are consistent with the expected range of modern brachiopod calcite and fall along the Global Brachiopod Mg Line^20^ (Supplementary Fig. 1a).

### Bulk and clumped isotope analyses

The δ^18^O_VPDB_ values of the modern brachiopod shells analysed in this study range between −2.20(±0.02)‰ and 3.92(±0.02)‰, while the δ^13^C_VPDB_ values range between −0.88(±0.02)‰ and 2.44(±0.01)‰. These values are consistent with the range in isotope compositions of modern brachiopod shells (secondary and tertiary layers) reported elsewhere^20,21,25,27^. The difference between the oxygen and carbon isotope composition of the shells determined using the [*Gonfiantini*] and the [*Brand*] sets of isotopic parameters^28^ is ~0.01‰. This is similar or less than the 1σ S.E. of replicate measurements and can therefore be ignored (see Methods).

The ∆_47(CDES 25)_ values measured for the modern brachiopods shells calculated with the [*Gonfiantini*] parameters range between 0.671(±0.007)‰ and 0.775 (±0.004)‰, while the 1σ S.E., calculated from 4–10 replicate analyses, ranges between 0.004–0.014‰. The ∆_47(CDES 25)_ values for the brachiopods calculated with the [*Brand*] parameters are between 0.664(±0.007)‰ and 0.767(±0.004)‰, while the 1σ S.E., calculated from 4–10 replicate analyses, ranges between 0.004–0.013‰. The difference between ∆_47(CDES 25)_ values calculated with the [*Gonfiantini*] and the [*Brand*] sets of isotopic parameters is between 0.005‰ and 0.008‰.

### Apparent ∆_47_–temperature relationship

**Figure 1:**
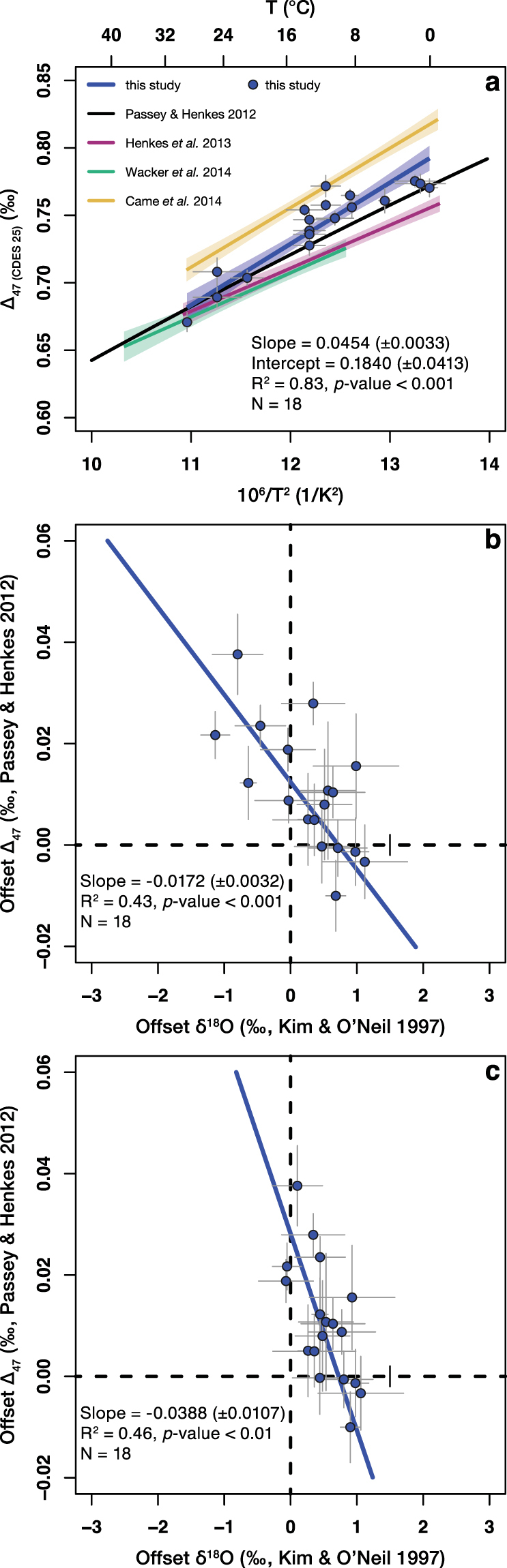
Brachiopods show an offset from equilibrium ∆_47_ and δ^18^O values. (**a**) ∆_47_–temperature dependence derived from the eighteen modern brachiopods analysed in this study, calculated using the [*Gonfiantini*] set of isotopic parameters. (**b**) The offset δ^18^O and offset ∆_47_ values show a significant negative correlation. The brachiopods, which show apparent clumped isotope equilibrium are enriched by up to +1‰, relative to Kim and O’Neil^35^. Seawater δ^18^O values were acquired from the Global Seawater Oxygen-18 Database^34^. (**c**) The correlation between offset δ^18^O and offset ∆_47_ values is still present if, where available, the directly measured seawater δ^18^O values (Supplementary Table 1) were used for the calculations. For all plots: linear regression lines fitted to our data consider the errors. Corresponding two-tailed *p*-values are computed using a t-test. Error bars for the offset δ^18^O values indicate the mean deviation from oxygen isotope equilibrium calculated using the minimum and the maximum temperature estimates. Error bars for the offset ∆_47_ values indicate the 1σ S.E. of the replicate measurements.

To obtain a ∆_47_–temperature relationship for modern brachiopod calcite, a least-squares fit linear regression^29,30^ was performed on the measured ∆_47(CDES 25)_ values and the independently-sourced brachiopod growth temperatures^31^ (Supplementary Tables 1 and 2). This approach considered uncertainties arising from both the clumped isotope measurements and the growth temperatures. The statistical analyses yielded the following ∆_47_–temperature relationship (Fig. 1a and Supplementary Fig. 2a):

1 $$\begin{array}{c}{{\rm{\Delta }}}_{47({\rm{C}}{\rm{D}}{\rm{E}}{\rm{S}}25)}=0.0454(\pm 0.0033)\times {10}^{6}/{{\rm{T}}}^{2}+0.1840(\pm 0.0413)\\ \quad \quad \quad {{\rm{R}}}^{2}=0.83,{p}-{\rm{v}}{\rm{a}}{\rm{l}}{\rm{u}}{\rm{e}} < 0.001,{\rm{N}}=18,[Gonfiantini]\end{array}$$

2 $$\begin{array}{c}{{\rm{\Delta }}}_{47({\rm{CDES}}25)}=0.0453(\pm 0.0033)\times {10}^{6}/{{\rm{T}}}^{2}+0.1789(\pm 0.0415)\\ \quad \quad \quad {{\rm{R}}}^{2}=0.83,p-\mathrm{value} < 0.001,{\rm{N}}=18,[Brand]\end{array}$$

where ∆_47_ is in ‰, T (temperature) is in K and the two-tailed *p*-values are calculated using a t-test.

The slope of our ∆_47_–temperature calibration line (eqs 1 and 2) is steeper compared to the theoretical calibration^10^ and to previous calibrations made at > 70 °C^11,29,32^, and shallower than most 25 °C calibrations^33^. However, the slope of our ∆_47_–temperature calibration line is indistinguishable from the brachiopod-only calibration of Came *et al*.^9^, made at 25 °C.

### ∆_47_ and δ^18^O offsets from apparent equilibrium

The difference between the measured and apparent equilibrium values are here referred to as offset values (Supplementary Data 2). Annual mean habitat temperatures (i.e., brachiopod growth temperatures) were acquired from the World Ocean Atlas 2013^31^. Annual mean seawater δ^18^O values representative of the sampling location and depth were taken from the Global Seawater Oxygen-18 Database^34^. For thirteen out of eighteen specimens, directly measured seawater δ^18^O values were also available, giving actual seawater oxygen isotope compositions at the water depth where the brachiopods were collected^20^ (Supplementary Table 1). To remain consistent, we distinguish between the two datasets: one using only the seawater δ^18^O values acquired from the Global Seawater Oxygen-18 Database^34^ and the other in which gridded δ^18^O values were replaced by the directly measured δ^18^O values where available.

Apparent δ^18^O_calcite_ equilibrium values were calculated using the 1000lnα_calcite–water_–temperature relationship of Kim and O’Neil^35^ (see Supplementary Information) and that of Brand *et al*.^20^, respectively. The latter includes a correction for the Mg-effect, which accounts for a 0.17‰ change per mol% MgCO_3_ in the δ^18^O values of the calcite, in agreement with laboratory precipitation experiments^20^. Apparent ∆_47_ equilibrium values were calculated using the theoretical calibration of Passey and Henkes^10^, i.e., their eq. 5, with the empirically determined intercept of 0.280 (Fig. 1b,c and Supplementary Fig. 2). This equation considers a 25–90 °C acid fractionation factor of 0.081‰ and has been verified in the 0–40 °C temperature range by empirical and experimental approaches^11,29,36,37^. In addition, offset ∆_47_ values were also calculated assuming that the most recent calibrations of Bonifacie *et al*.^32^ or Kelson *et al*.^38^ represent the clumped isotope equilibrium (Supplementary Fig. 3). Offset ∆_47_ values were computed using both the [*Gonfiantini*] and the [*Brand*] processed data.

Most of the analysed brachiopods in this study exhibit combined offsets from clumped and oxygen isotope equilibrium, irrespective how the offset values were calculated (Fig. 1b,c and Supplementary Figs 2b,c and 3). The largest deviations from the equilibrium ∆_47_ values were observed in the temperate- to cold-water brachiopod species, particularly those of the species *Magellania venosa* and *Magasella sanguinea*. In contrast, most of the warm-water (>20 °C) taxa, such as *Thecidellina congregata*, *Argyrotheca* sp., *Megerlia* sp., and *P*. *atlantica*, exhibit apparent clumped isotope equilibrium.

### Oxygen isotope analyses with ion probe

**Figure 2:**
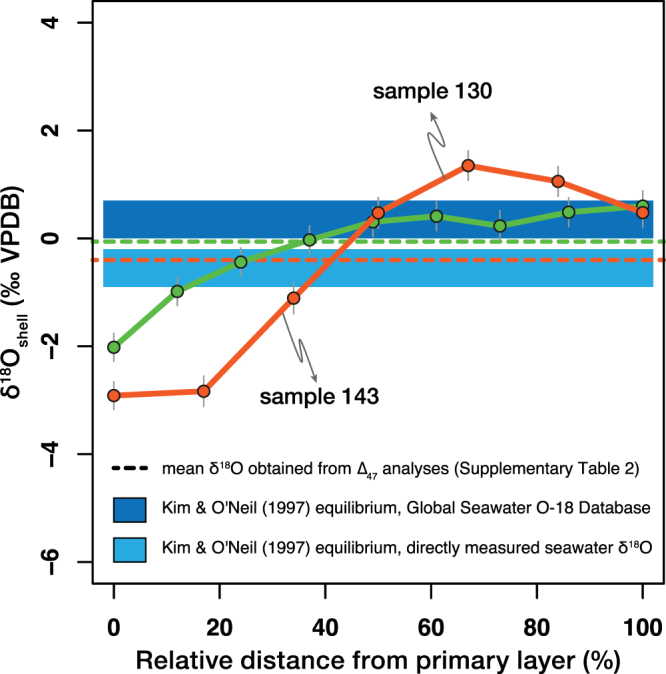
Variations in δ^18^O between the outer and the inner part of the secondary layer. Results of the SIMS transects made on two *M*. *venosa* shells (samples 130 and 143) also analysed for clumped isotopes. Apparent equilibrium ranges for δ^18^O were calculated according to Kim and O’Neil^35^ using the minimum and maximum habitat temperature estimates and the two sets of seawater δ^18^O values (see main text and Supplementary Table 1). Error bars for the δ^18^O indicate the external reproducibility (1σ S.D.) based on replicate measurements of carbonate standards.

High resolution (20 μm) *in situ* oxygen isotope analysis was performed on two *M*. *venosa* shells using SIMS (Secondary Ion Mass Spectrometry). In the secondary layer of the two investigated *M*. *venosa* shells, the δ^18^O values range from −2.91‰ to 1.35‰ (sample 130) and from −2.02‰ to 0.60‰ (sample 143; Fig. 2). This species does not have a tertiary layer and the primary layer was too thin to be analysed (Supplementary Fig. 6). The variation in δ^18^O between the outer and inner part of the shell was 4.3‰ for sample 130 and 2.6‰ for sample 143 (Supplementary Data 3, Fig. 2).

## Discussion

Multiple processes can lead to the deviation of measured δ^18^O and ∆_47_ values from thermodynamic equilibrium. If the offset seen in the modern brachiopod δ^18^O values would arise solely from the varying Mg-content of the analysed shells, one would expect that this offset would disappear if the equilibrium values were calculated using the equation of Brand *et al*.^20^ instead of Kim and O’Neil^35^, since the former includes a correction for the Mg-effect. However, the scatter of the brachiopod δ^18^O values around the assumed oxygen isotope equilibrium is even greater if the Brand *et al*.^20^ equation is used, thus the offset δ^18^O values cannot be explained by the Mg-content of the shells (compare Fig. 1b,c to Supplementary Figs. 1b,c). We also exclude a direct effect of the Mg-content of the shells on the ∆_47_ values, considering the recent findings of Bonifacie *et al*.^32^ who, for a given precipitation temperature, did not find any difference in the clumped isotope composition between dolomite and calcite reacted at 90 °C.

A mixture of carbonates of different compositions will mix linearly with respect to δ^13^C and δ^18^O but non-linearly with respect to ∆_47_. The resulting mixture can, therefore, have a greater or lower ∆_47_ value than the weighted sum of the end-member ∆_47_ values, introducing an artificial bias in ∆_47_^39,40^. The range of variation in δ^18^O and δ^13^C in modern brachiopod shells is usually not larger than 6‰, considering both the variation within secondary layer calcite^19,21–24,41^ and between the juvenile and the adult parts of the shell^21,22,24–27,41–43^. Assuming the most-extreme scenario of 50–50% mixing of carbonates precipitated at the same temperature with a 6‰ difference in both their δ^13^C and δ^18^O values, the maximum effect of carbonate mixing on ∆_47_ in modern brachiopods would be +0.009‰^40^. To further investigate the role of sample heterogeneity on our data, we assessed the range of variation in δ^18^O values in two *M*. *venosa* shells that exhibited a high (0.024‰ and 0.038‰, respectively) ∆_47_ offset with respect to Passey and Henkes^10^. Our data show that the difference in δ^18^O values in brachiopod secondary layer calcite can be as high as 4.3‰ between the outer and the inner part of the shell (Fig. 2). A covariance of δ^18^O and δ^13^C along the depth transects of modern brachiopods shells suggests that the range of variation in δ^13^C will be comparable to that of δ^18^O^22–24,41,43^. A 50–50% mixing of carbonates precipitated at the same temperature with a 4.3‰ difference in both their δ^13^C and δ^18^O values results in a ∆_47_ mixing effect of +0.005‰^40^. This bias is much smaller than the observed maximum offset ∆_47_ value of 0.038‰ and unresolvable from the external analytical precision (1σ S.E.) received for most replicates. Thus, it is highly unlikely that a mixing of carbonates of different compositions through the shell significantly contributes to the positive ∆_47_ offsets observed in this study.

Differences in laboratory procedures, such as reaction temperatures, have previously been suggested as a possible explanation for the discrepancy in the slopes between the Came *et al*.^9^ calibration, made at 25 °C, and the Henkes *et al*.^11^ calibration, made at 90 °C. Although, Henkes *et al*.^11^ also analysed brachiopods (N = 4), their calibration is predominantly based on molluscs (N = 40). Since our study and that of Came *et al*.^9^ yielded the same calibration slope despite using two different acid digestion temperatures, we consider this slope gradient to be characteristic of brachiopods and exclude acid digestion temperature as a valid explanation for the difference between the lower (25 °C) and the higher (>70 °C) temperature calibration slopes.

**Figure 3:**
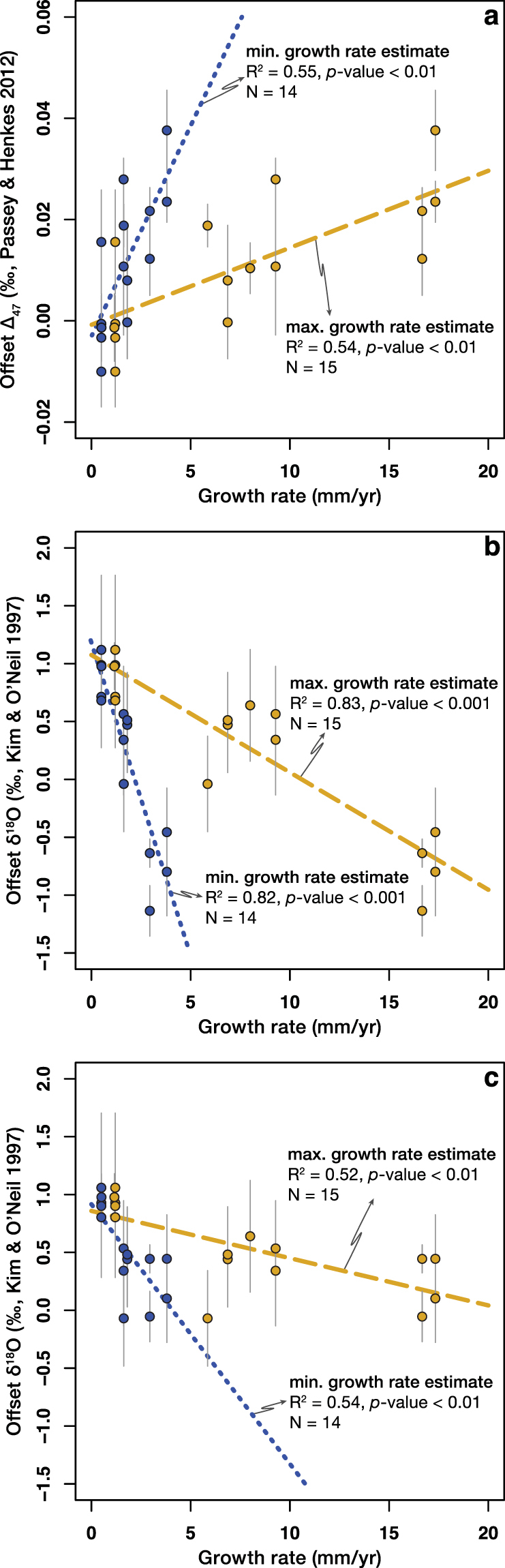
Offset ∆_47_ and offset δ^18^O values correlate with brachiopod growth rates. The dashed and dotted lines are simple linear regressions calculated using the maximum and the minimum growth rate estimates, respectively. (**a**) Offset ∆_47_ values, calculated using the [*Gonfiantini*] set of isotopic parameters,﻿ positively correlate with brachiopod growth rates. (**b**) Offset δ^18^O values negatively correlate with brachiopod growth rates. Seawater δ^18^O values were acquired from the Global Seawater Oxygen-18 Database^34^. (**c**) The correlation between offset δ^18^O and brachiopod growth rates is still present if, where available, the directly measured seawater δ^18^O values were used for the calculations. For all plots: two-tailed *p*-values are calculated using a t-test. Error bars for the offset δ^18^O values indicate the mean deviation from oxygen isotope equilibrium calculated using the minimum and the maximum temperature estimates. Error bars for the offset ∆_47_ values indicate the 1σ S.E. of the replicate measurements.

Growth rates of the modern brachiopods analysed in this study correlate well with both the offset ∆_47_ (R^2^ ≈ 0.55, *p*-value < 0.01; Fig. 3a) and the offset δ^18^O values (R^2^ > 0.52, *p*-value < 0.01; Fig. 3b,c). The slowest growing brachiopods (*T*. *congregata*, *Argyrotheca* sp., *Megerlia* sp., *P*. *atlantica*) are in apparent clumped isotope equilibrium^10^, whereas the fastest growing brachiopods (*M*. *venosa*, *T*. *transversa*) show a positive ∆_47_ offset. The offset δ^18^O values show a negative correlation with growth rate. A similar depletion of ^18^O with increasing growth rate was observed in other modern brachiopod species by Takayanagi *et al*.^44^. The slowest growing brachiopods that are closest to clumped isotope equilibrium relative to Passey and Henkes^10^ are enriched in ^18^O relative to the apparent oxygen isotope equilibrium as predicted by Kim and O’Neil^35^. This strongly implies that growth rate exerts control on the kinetic mechanisms responsible for the observed departures from apparent clumped and oxygen isotope equilibria.

The calcite shells of articulated brachiopods are secreted in the outer epithelium of the mantle. The primary layer is formed by indirect secretion in the extrapallial fluid, while the secondary layer is formed by extracellular mineralization^45,46^. To aid our discussion, we consider a simplified model of calcification, introduced for molluscs and corals, but that has also been applied to brachiopods^47,48^ (Supplementary Fig. 4a). In this model, carbonate formation occurs in a semi-isolated volume, separated from the ambient environment by an organic membrane^13,46,49^. The organism requires calcium (Ca^2+^) and carbonate (CO_3_^2−^) ions to enable the precipitation of CaCO_3_. In marine calcifiers, such as corals and molluscs, the organic membrane pumps Ca^2+^ into the calcifying fluid using an enzyme (Ca-ATPase), while increasing the pH of the fluid by removing an equivalent number of protons (2H^+^)^13^. We note that the presence of this enzyme, to the best of our knowledge, has not been reported from brachiopods to date. As the membrane is only permeable for aqueous carbon dioxide (CO_2(aq)_), the CO_2(aq)_ in the mineralizing fluid is transformed into bicarbonate (HCO_3_^−^) and CO_3_^2−^ ions via hydration (eq. 3) and hydroxylation (eq. 4) reactions:

3 $${{\rm{CO}}}_{2(aq)}+{{\rm{H}}}_{2}{\rm{O}} < \mbox{--} > {{\rm{H}}}^{+}+{{{\rm{HCO}}}_{3}}^{-}$$

4 $${{\rm{CO}}}_{2(aq)}+{{\rm{OH}}}^{-} < \mbox{--} > {{{\rm{HCO}}}_{3}}^{-}$$

It has recently been demonstrated that kinetic effects related to the CO_2(aq)_ hydration and hydroxylation reactions, as well as diffusion, can produce a positive ∆_47_ and a negative δ^18^O offset from thermodynamic equilibrium^12,50,51^ (Supplementary Fig. 4b).

Knudsen-diffusion predicts that a gas diffusing through a membrane will be depleted in ^18^O but enriched in ∆_47_, relative to the residual gas^51^. When correlating the ∆_47_ and δ^18^O offsets from equilibrium, the diffused and residual gas fractions would plot along a slope of −0.023. An identical slope would be obtained if kinetic fractionations were evoked by diffusion of CO_2(aq)_ through water^51^.

The reaction rate of hydration and hydroxylation of the dissolved CO_2_ is orders of magnitude slower than the reaction rate of bicarbonate dissociation^52^. Both CO_2(aq)_ hydration and hydroxylation preferentially select light isotopes (^16^O, ^12^C) and discriminate against heavy isotopes (^18^O, ^13^C). If the carbonate precipitation rate is high, HCO_3_^−^ can dissociate into CO_3_^2−^ and H^+^ before reaching equilibrium with CO_2(aq)_ in the calcifying fluid, therefore, the solid carbonate will inherit lighter δ^18^O values^49,53^. Simultaneously, incomplete CO_2(aq)_ hydration or hydroxylation results in an increased ∆_47_ value of the aqueous HCO_3_^−^, which can be inherited by the solid carbonate if the precipitation rate is high^14^. Theoretical calculations predict a regression slope between the offset ∆_47_ offset and offset δ^18^O values in the order of −0.05 and −0.01 for kinetic controls associated with hydration and hydroxylation reactions, respectively^12,54^ (Supplementary Fig. 4b). Carbonic anhydrase, an enzyme often present in calcifying organisms, such as corals, promotes rapid oxygen isotope exchange between dissolved inorganic carbon species^55,56^. If this enzyme was present in brachiopods, it could reduce or eliminate the kinetic isotope effects caused by the slow hydration and hydroxylation reactions. However, carbonic anhydrase has not been identified in the calcifying fluid of modern brachiopods^57^.

Our data exhibits an offset ∆_47_–δ^18^O slope of −0.017(±0.03) if the offset δ^18^O values are calculated using the seawater oxygen isotope compositions acquired from the Global Seawater Oxygen-18 Database^34^ (Fig. 1b). If the offset δ^18^O values are calculated using directly measured water δ^18^O values where possible, the offset ∆_47_–δ^18^O slope becomes as steep as −0.039(±0.01) (Fig. 1c). Both slopes are significant and stay consistent, irrespective of data processing, i.e., [*Gonfiantini*] *vs*. [*Brand*] parameters, and the clumped isotope calibration we used to calculate the offset values (Supplementary Figs 2b,c and 3).

**Figure 4:**
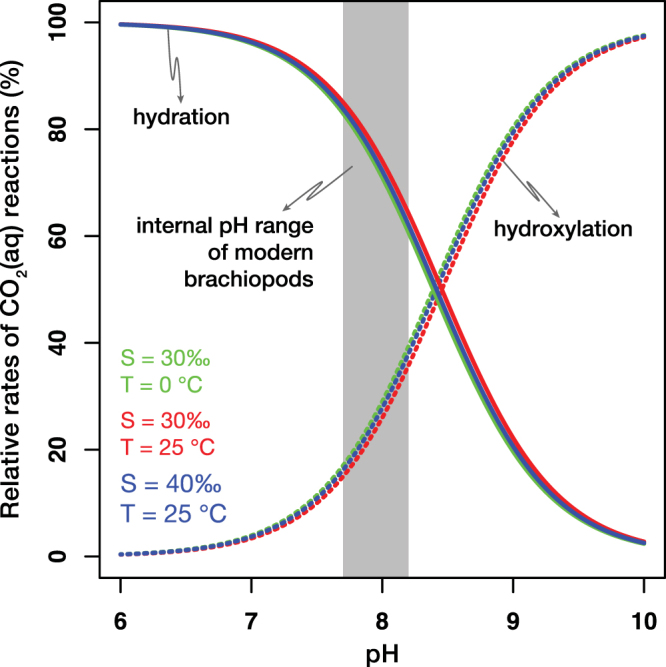
At conditions characteristic to modern seawater, hydration is the dominant reaction at which CO_2(aq)_ transforms into HCO_3_^−^ in the brachiopod calcifying fluid. The balance between the rates of hydration and hydroxylation reactions is defined as R = (k_CO2_/(k_CO2_ + k_OH_/*a*H) * 100, where k_CO2_ and k_OH_ are the rate constants for CO_2(aq)_ hydration and hydroxylation, respectively and *a*H is the H^+^ activity^52^. R is calculated for three salinity (S) – temperature (T) scenarios, characteristic to modern seawater. The shaded area represents the internal pH range of modern brachiopods^41^.

The observed correlation slopes (−0.017 to −0.039, depending on the seawater δ^18^O dataset) between offset ∆_47_ and offset δ^18^O point to the importance of kinetic effects associated with diffusion and incomplete hydration and hydroxylation of CO_2(aq)_. The hydration and hydroxylation reactions occur superimposed onto the diffusion of CO_2(aq)_. Consequently, diffusion alone cannot be the sole kinetic mechanism responsible for the observed trend in our data. If heterogeneous oxygen isotope exchange between water and CO_2(aq)_ proceeds to equilibrium, it would erase the offsets from equilibrium ∆_47_ and δ^18^O generated during diffusion. Tang *et al*.^37^ precipitated calcites at a pH < 9 and >10 and observed that ∆_47_ values increased by approximately 0.016‰ for every 1‰ decrease in δ^18^O at high (>10) pH. They suggested that a combined effect of diffusion and hydroxylation could be responsible for the observed slope. Interestingly, their slope is indistinguishable from the one we observe when we exclusively use the Global Seawater Oxygen-18 Database^34^ to infer seawater δ^18^O. However, pH > 10 would be inconsistent with the internal pH range of modern brachiopods, which is likely to be between 7.7 and 8.2, calculated from δ^11^B values^41^. Hydration is more prominent at low (<8.4) pH while hydroxylation is more prominent at high (>8.4) pH, assuming temperature and salinity values characteristic of modern seawater^49,52^ (Fig. 4). In the pH range characteristic for modern brachiopods, only 10–40‰ of the oxygen isotope exchange between water and CO_2(aq)_ should occur via the hydroxylation reaction, whereas 60–90% should proceed via the hydration reaction^52^ (Fig. 4). Such a dominance of hydration over hydroxylation would result in an offset ∆_47_–δ^18^O slope of −0.034 to −0.046, assuming estimated slopes of −0.05 and −0.01 to be characteristic of the hydration and hydroxylation reactions, respectively^12,54^. This range of slopes is in agreement with our result based on the dataset that combines gridded seawater δ^18^O data with directly measured values (Fig. 1c and Supplementary Figs 2c and 3b,d).

**Figure 5:**
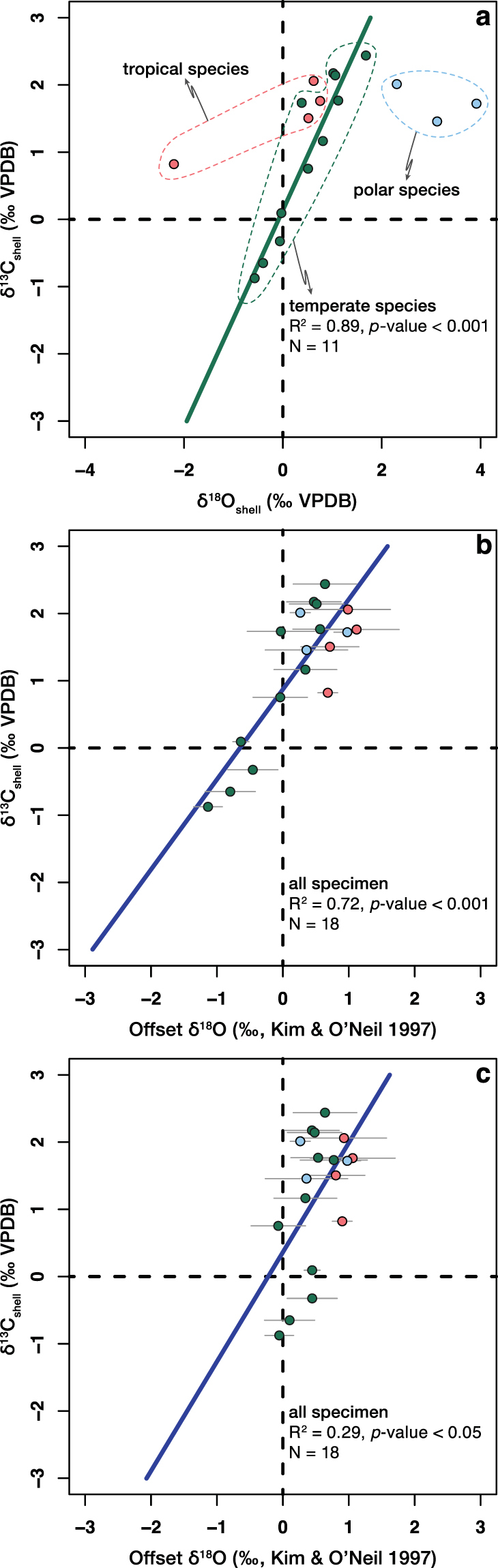
Correlation between brachiopod shell bulk oxygen and carbon isotope compositions. (**a**) The shell δ^18^O values of the temperate modern brachiopods analysed in this study positively correlate with corresponding δ^13^C values. (**b**) Shell δ^13^C values positively correlate with offset δ^18^O values for all modern brachiopods analysed in this study. Seawater δ^18^O values were acquired from the Global Seawater Oxygen-18 Database^34^. (**c**) The correlation between shell δ^13^C and offset δ^18^O is still present if, where available, the directly measured seawater δ^18^O values were used for the calculations. For all plots: two-tailed *p*-values are calculated using a t-test for the simple linear regressions. Error bars for the offset δ^18^O values indicate the mean deviation from oxygen isotope equilibrium calculated using the minimum and the maximum temperature estimates.

The δ^18^O values of the temperate modern brachiopod shells analysed in this study correlate well with corresponding δ^13^C values (R^2^ = 0.89, *p*-value < 0.001; Fig. 5a). Brand *et al*.^58^ showed that parallel correlations are present between shell δ^18^O and δ^13^C among tropical (latitudes < 30°), temperate (latitudes 30°–60°) and polar (latitudes > 60°) brachiopods, respectively. These parallel trends are related to the distinct habitat seawater temperatures and oxygen isotopic compositions of these groups^58^. With the exchange of the shell δ^18^O values for the offset δ^18^O values, the seawater and temperature effect on the shell δ^18^O can be eliminated. The offset δ^18^O values of the modern brachiopod shells analysed in this study correlate with corresponding δ^13^C values (R^2^ > 0.29, *p*-value < 0.05; Fig. 5b,c). A covariation of δ^18^O and δ^13^C has been also observed in single brachiopod shells by other authors^22–24,41,43^. A synchronous depletion of the heavy isotopes (^18^O and ^13^C) in biogenic carbonates, as observed for the modern brachiopods analysed in this study (Fig. 5a,b,c), agrees with the preferential selection of light isotopes during CO_2(aq)_ hydration and hydroxylation reactions^49,53,59^.

There is still an ongoing debate if the experiments of Kim and O’Neil^35^ are characteristic for the attainment of overall equilibrium between calcite and water. A natural example for equilibrium precipitation might be the Devil’s Hole carbonate that grew extremely slowly in a constant geochemical environment. Its isotope composition has therefore been postulated not to be affected by kinetics^60^. The δ^18^O value of the Devil’s Hole carbonate is approximately +1.5‰ higher than could be calculated using the equation of Kim and O’Neil^35^. Laboratory experiments, comparing oxygen isotope fractionation factors of slowly and rapidly precipitated synthetic calcites^61^, and theoretical computations^62^ provide further evidence that the Kim and O’Neil^35^ equation is not representative of thermodynamic equilibrium between calcite and water. The δ^18^O values of the modern brachiopods analysed in this study, which show apparent clumped isotope equilibrium, are enriched by up to 1‰, relative to the Kim and O’Neil^35^ equilibrium (Fig. 1b,c and Supplementary Figs 2b,c and 3). This finding is in line with the hypothesis^60–62^ that oxygen isotope equilibrium between calcite and water is expressed by fractionations exceeding those of Kim and O’Neil^35^.

In summary, the oxygen and clumped isotope composition of modern brachiopod shells are affected by growth rate-induced kinetic effects (i.e., incomplete hydration and/or hydroxylation of CO_2(aq)_ at higher growth rates) as indicated by (1) the negative correlation between offset ∆_47_ and offset δ^18^O values, (2) the correlation between growth rates and both ∆_47_ and δ^18^O offsets and (3) the positive correlation between shell δ^13^C and δ^18^O values. Kinetic effects may significantly contribute to the bottom seawater temperatures calculated from brachiopod shell δ^18^O and ∆_47_. Combining isotope data derived from multiple species is likely to result in higher variability in observed δ^18^O and ∆_47_ that does not reflect real temperature changes. Based on our findings, information about taxon-specific growth rate and kinetics involved in calcite precipitation is essential whenever constraining seawater temperatures from ∆_47_ and δ^18^O values in brachiopods. A future study considering seasonal variations in seawater δ^18^O values and in growth rates could further improve our understanding of the nature and extent of the kinetic isotope effect in brachiopods.

## Methods

### Sampling of shells

Organic tissue and encrusting organisms were removed from the brachiopod shells using a metal pin and a brush. Specimen S006L, DS420L, and DS430L were submerged in diluted NaOCl for 5–10 minutes to soften the organic material. The shells were cleaned in an ultrasonic bath using deionized water. Afterwards, the shells were dried using pressured air and stored at room temperature. For the larger species, the primary layer of the shells was mechanically removed using a hand-held electric drill (Proxxon Micromot IBS/E) on the lowest speed setting and only the secondary and/or tertiary layers were sampled. An approximately 0.5 cm^2^ area from the anterior part of the ventral valve was crushed and homogenised using an agate mortar and pestle. We avoided sampling the umbo area, the hinge area, the muscle scar area and the youngest parts of the shell. Exceptions to this were the micromorphic shells of *P*. *atlantica*, *T*. *congregata*, *Argyrotheca* sp., and *Megerlia* sp., where 4 to 20 whole shells had to be crushed to acquire enough material for multiple replicate analyses.

### Growth rate

The growth of brachiopods can be described by the von Bertalanffy asymptotic function^63^. Juvenile individuals grow the fastest and the growth rate decreases with age, before reaching the species-specific maximum size. To acquire comparable, species-specific growth rates, we estimated a minimum and a maximum growth rate for each analysed species. The maximum growth rate, in our case, depicted the average growth rate of the brachiopod until it reached 50% of its maximum size. Similarly, the minimum estimate was the average growth rate after the brachiopod had already reached 50% of its maximum size. For *M*. *venosa*^63^, *M*. *fragilis*^64^, *M*. *sanguinea*^65^, *C*. *inconspicua*^66^, *T*. *transversa*^67^ and *L*. *neozelanica*^68^, detailed studies were available, thus both a maximum and a minimum growth rate estimate could be calculated. Such a study has not, to date, been made for *N*. *nigricans*, therefore we used the available average juvenile growth rate^69^ as a maximum estimate. For the micromorphic brachiopods *P*. *atlantica*, *Argyrotheca* sp., *Megerlia* sp., and *T*. *congregata*, we assumed a 0.5 mm/yr and a 1.2 mm/yr as a minimum and as a maximum growth rate estimate, respectively. These growth rates are characteristic for micromorphic brachiopods^70^.

### Trace element analyses

The magnesium content of the studied brachiopod shells was analysed using a Thermo Scientific iCap 6000 dual view ICP-OES (Inductively Coupled Plasma - Optical Emission Spectrometry) at the Goethe University, Frankfurt, Germany. For the analyses, we took 120–150 μg of carbonate powder from the homogenised batches that were also used for the isotope measurements and dissolved them in 0.500 cm^3^ 2% HNO_3_. An aliquot of 0.300 cm^3^ of the sample solution was diluted with 1.500 cm^3^ yttrium water (until 1.000 mg/dm^3^) prior to measurement to correct for matrix biases during analyses. The Mg/Ca measurements were drift-corrected and standardized to an internal consistency standard (ECRM 752–1) measured alongside with the samples. The external reproducibility (2σ S.E.) for this standard was ±0.1 mmol/mol Mg/Ca. Finally, the MgCO_3_ concentration values (mol%) were adjusted to a 100% carbonate basis and were normalised to a combined Ca and Mg value of 395,000 ppm^20^.

### Stable isotope analyses

Clumped isotope analyses were made using a fully automated gas extraction and purification line connected to a ThermoFisher MAT 253 gas-source isotope-ratio mass spectrometer at the Goethe University, Frankfurt, Germany. Homogenised carbonate powder was reacted at 90 °C with >105% phosphoric acid. In general, six replicate analyses were made every day including one carbonate standard, one equilibrated gas (1000 °C or 25 °C) and four sample replicates. Background correction was performed for the sample and the reference gas separately, as described in Fiebig *et al*.^71^ (Supplementary Fig. 5). Background-corrected equilibrated gas data displays slopes that are within errors indistinguishable from zero (Supplementary Data 1). Additional information concerning the methodology of the clumped isotope measurements can be found in the Supplementary Information.

The raw ∆_47_, δ^18^O and δ^13^C values were calculated using two sets of isotope parameters^28^. In the [*Gonfiantini*] set, the parameters are as follows: R^13^_PDB_ = 0.0112372, R^18^_VSMOW_ = 0.0020052, R^17^_VSMOW_ = 0.0003799 and λ = 0.5164. In the [*Brand*] set, the parameters are: R^13^_PDB_ = 0.01118, R^18^_VSMOW_ = 0.0020052, R^17^_VSMOW_ = 0.00038475 and λ = 0.528. The raw ∆_47_ values were projected to the CDES (Carbon Dioxide Equilibrium Scale^72^) using equilibrated gases. Empirical transfer functions (ETFs) were determined using gases of various bulk isotope compositions equilibrated at 25 °C and at 1000 °C, respectively. The intercept values of the equilibrated gases in the ∆_47_–δ_47_ space were constant between 06.06.2016–12.22.2016 and 01.06.2017–04.05.2017 (Supplementary Data 1, Supplementary Table 3). For referencing the ∆_47_ values to 25 °C, we used an acid fractionation factor of 0.081‰^10^. Two internal carbonate reference materials were analysed along with the samples to verify the precision and the stability of clumped isotope measurements: Carrara marble calcite (Carrara) and *Arctica islandica* (mollusk) shell aragonite (MuStd). The mean ∆_47(CDES 25)_ values (±1σ S.E.) for the Carrara (N = 123) and the MuStd (N = 83) reference materials, calculated using the [*Gonfiantini*] set of isotopic parameters were 0.396(±0.001)‰ and 0.743(±0.002)‰, and using the [*Brand*] set of isotopic parameters were 0.395(±0.001)‰ and 0.738(±0.002)‰, respectively. The 1σ S.D. of the ∆_47_ values for the reference materials is 0.014‰. The [*Gonfiantini*] values agree within ≤ 0.005‰ with corresponding ∆_47(CDES 25)_ values reported elsewhere for Carrara^11,32,72^ and MuStd^73^ after applying a consistent acid fractionation factor to these datasets.

### Secondary Ion Mass Spectrometry

The ion probe analyses were carried out using a Caméca IMS 1280-HR2 at CRPG-CNRS (Nancy, France). A short summary of the technique is reported in the Supplementary Information. The exact location of the ion probe transects and the analysed points are shown on Supplementary Figure 6. The two analysed shells were also investigated for clumped isotopes. The ventral valve of each brachiopod was cut in half from anterior to posterior part to produce a longitudinal section. One half was mounted in epoxy and polished with diamond paste down to 1µm. Transects from the outermost (primary layer) to the innermost part (closest to mantle cavity) of the shell were performed with 20 µm spots and with a constant step of 50 µm. The number of analysis was determined by the shell thickness. The location of the transect was approximately 3 mm above the anterior margin, at the exact location where the shell was sampled for the clumped and trace element analyses.

### Data availability

All data pertinent to this manuscript and its reported findings can be found in the manuscript itself or the associated Supplementary Information file.

## Electronic supplementary material

Supplementary Information

Supplementary Data 1

Supplementary Data 2

Supplementary Data 3
