# Supplementary material for: Assessing kinetic fractionation in brachiopod calcite using clumped isotopes
Source: Sci Rep. 2018 Jan 11;8:533. doi: 10.1038/s41598-017-17353-7 (PMC5765054; doi:10.1038/s41598-017-17353-7)
Supplement: Supplementary file 1 — Supplementary Information [file 41598_2017_17353_MOESM1_ESM.pdf]

Supplementary Information to:

Assessing kinetic fractionation in brachiopod calcite using clumped isotopes

David Bajnai; Jens Fiebig; Adam Tomašových; Sara Milner Garcia; Claire Rollion-Bard; Jacek Raddatz; Niklas Löffler; Cristina Primo-Ramos; Uwe Brand

SUPPLEMENTARY TABLE 1 2
SUPPLEMENTARY TABLE 2 3
SUPPLEMENTARY FIGURE 1 4
SUPPLEMENTARY FIGURE 2 5
SUPPLEMENTARY FIGURE 3 6
SUPPLEMENTARY FIGURE 4 7
CLUMPED ISOTOPE ANALYSES 8
SUPPLEMENTARY TABLE 3 8
SUPPLEMENTARY FIGURE 5 9
OXYGEN ISOTOPE ANALYSES WITH ION PROBE (SIMS) 10
SUPPLEMENTARY FIGURE 6 10
OXYGEN ISOTOPE FRACTIONATION FACTORS 11
REFERENCES 12

Supplementary Data

(in separate Excel files)

Supplementary Data 1.xlsx .... Results of the clumped isotope measurements. This file contains all replicate analyses for the samples, standards and equilibrated gases, including the dates of the measurements as well as the ETFs.

Supplementary Data 2.xlsx .... A summary table for the results of the stable isotope and trace element analyses and for the environmental and growth parameters of the modern brachiopods investigated in this study. This file contains calculations for the offset Δ_{47} and offset $\delta^{18}O$ values.

Supplementary Data 3.xlsx .... A summary table for the SIMS analyses.

Supplementary Table 1 | Environmental and growth parameters of the modern brachiopods investigated in this study										
**Sample**	**Species**	**Location (Lat., Long.)**	**Water depth (m)**	**$\delta^{18}O_{water}$ (‰ VSMOW)**	**Temperature (°C, annual)**	**Temperature (°C, max.)**	**Temperature (°C, min.)**	**Growth rate (mm/yr, max.)**	**Growth rate (mm/yr, min.)**	**Valve length (mm, ventral)**
130	*Magellania venosa*	Lilliguapi, Chile<br>-42.162030, -72.598580	18	-0.31<br>-1.21*	11.36	13.19	9.77	17.3	3.8	64.7
143	*Magellania venosa*	Punta Gruesa, Chile<br>-42.409833, -72.424333	20	-0.31<br>-1.21*	11.36	13.19	9.77	17.3	3.8	55.9
ChHP1	*Hemithiris psittacea*	Churchill, Canada<br>58.786867, -94.175450	20	-2.55<br>-3.35*	1.56	3.20	-1.05	-	-	16.8
D487L	*Terebratalia transversa*	San Juan Is., WA, USA<br>48.4965, -122.947	64	-0.76<br>-1.84*	8.59	9.62	7.70	16.7	2.9	21.8
DA5.25.1	*Argyrotheca sp.*	Dahab, Egypt<br>28.51, 34.52	10	1.80<br>1.86*	24.84	28.19	21.83	1.2	0.5	< 1
DA5.25.2	*Megerlia sp.*	Dahab, Egypt<br>28.51, 34.52	9	1.80<br>1.86*	24.84	28.19	21.83	1.2	0.5	< 1
DS288L	*Magasella sanguinea*	Doubtful Sound, NZ<br>-45.349, 167.0506	20	0.27<br>0.30*	13.27	15.44	11.68	9.3	1.6	33.4
DS420L	*Calloria inconspicua*	Doubtful Sound, NZ<br>-45.349, 167.0506	20	0.27<br>0.30*	13.27	15.44	11.68	5.9	1.6	21.4
DS430L	*Liothyrella neozelanica*	Doubtful Sound, NZ<br>-45.349, 167.0506	20	0.27<br>0.30*	13.27	15.44	11.68	6.9	1.8	51.5
DS431L	*Liothyrella neozelanica*	Doubtful Sound, NZ<br>-45.349, 167.0506	20	0.27<br>0.30*	13.27	15.44	11.68	6.9	1.8	45.8
FTD1	*Terebratella dorsata*	Falkland Islands<br>-53.0, -60.0	50-400	-0.17	4.76	5.59	4.29	-	-	26.3
GS183L	*Magasella sanguinea*	George Sound, NZ<br>-44.85, 167.35	18	0.31	13.85	16.57	12.20	9.3	1.6	29.6
NN2V	*Notosaria nigricans*	Kaka Point, NZ<br>-46.3866, 169.7823	2-15	0.10	10.31	12.72	8.41	8.0	-	14.5
PA.01	*Pajaudina atlantica*	La Palma, Canary Is.<br>28.455783, -17.846747	14	1.16<br>1.07*	20.92	23.24	19.01	1.2	0.5	2–6
S006L	*Terebratalia transversa*	San Juan Is., WA, USA<br>48.4919, -122.94945	73	-0.76<br>-1.84*	8.39	8.94	7.87	16.7	2.9	32.8
SAMID10116	*Glaciarcula spitzbergensis*	Svalbard, Norway<br>79.911, 15.812	46	-0.34	1.00	3.89	-1.32	-	-	10.2
TC.01	*Thecidellina congregata*	Rock Islands, Palau<br>7.272167, 134.380667	2	0.09<br>-0.13*	28.91	29.37	27.85	1.2	0.5	2–3
WMF1	*Magellania fragilis*	Weddell Sea, Antarctica<br>-69.950000, -11.816667	215	-0.39	0.06	0.87	-0.80	1.2	0.5	–

Ambient habitat temperatures for the studied brachiopods were acquired from the World Ocean Atlas 2013[1]. Mean temperatures depict the yearly average temperatures, while the minimum and maximum estimates are the mean monthly temperature of the coldest and the warmest month, respectively. Seawater $\delta^{18}O$ values were acquired either from the Global Seawater Oxygen-18 Database[2] or were measured directly[3] (marked with an asterisk*).

**Supplementary Table 2 | Results of the stable isotope and trace element analyses**						
**Sample**	**n**	**$MgCO_3$ (mol%)**	**Δ_{47} [*Gonfiantini*] (‰ CDES 25)**	**Δ_{47} [*Brand*] (‰ CDES 25)**	**$\delta^{18}O_{shell}$ [*Gonfiantini*] (‰ VPDB)**	**$\delta^{13}C_{shell}$ [*Gonfiantini*] (‰ VPDB)**
130	10	0.37 (±0.1)	0.772 (±0.008)	0.765 (±0.008)	-0.40 (±0.06)	-0.65 (±0.02)
143	10	0.36 (±0.1)	0.758 (±0.004)	0.751 (±0.004)	-0.06 (±0.04)	-0.32 (±0.01)
ChHP1	5	0.31 (±0.1)	0.775 (±0.004)	0.767 (±0.004)	0.38 (±0.01)	1.73 (±0.01)
D487L	6	0.34 (±0.1)	0.765 (±0.005)	0.757 (±0.005)	-0.57 (±0.02)	-0.88 (±0.02)
DA5.25.1	5	4.06 (±0.1)	0.708 (±0.010)	0.701 (±0.010)	0.62 (±0.03)	2.06 (±0.01)
DA5.25.2	4	4.11 (±0.1)	0.689 (±0.007)	0.684 (±0.007)	0.75 (±0.03)	1.76 (±0.02)
DS288L	6	0.67 (±0.1)	0.739 (±0.014)	0.732 (±0.013)	1.12 (±0.02)	1.77 (±0.01)
DS420L	6	0.55 (±0.1)	0.747 (±0.004)	0.740 (±0.004)	0.51 (±0.01)	0.75 (±0.01)
DS430L	6	0.67 (±0.1)	0.728 (±0.007)	0.721 (±0.007)	1.02 (±0.03)	2.17 (±0.02)
DS431L	6	0.55 (±0.1)	0.736 (±0.011)	0.729 (±0.010)	1.06 (±0.03)	2.14 (±0.01)
FTD1	4	0.51 (±0.1)	0.761 (±0.009)	0.754 (±0.009)	2.30 (±0.01)	2.01 (±0.01)
GS183L	5	0.45 (±0.1)	0.754 (±0.004)	0.748 (±0.004)	0.81 (±0.07)	1.17 (±0.01)
NN2V	6	0.65 (±0.1)	0.748 (±0.005)	0.741 (±0.005)	1.68 (±0.02)	2.44 (±0.00)
PA.01	5	6.80 (±0.1)	0.704 (±0.006)	0.698 (±0.006)	0.52 (±0.01)	1.51 (±0.01)
S006L	10	0.27 (±0.1)	0.756 (±0.007)	0.749 (±0.007)	-0.03 (±0.04)	0.09 (±0.03)
SAMID10116	4	0.45 (±0.1)	0.773 (±0.007)	0.767 (±0.007)	3.12 (±0.03)	1.46 (±0.01)
TC.01	5	5.24 (±0.1)	0.671 (±0.007)	0.664 (±0.007)	-2.20 (±0.02)	0.82 (±0.01)
WMF1	5	0.76 (±0.1)	0.770 (±0.007)	0.765 (±0.006)	3.92 (±0.02)	1.72 (±0.01)

The Δ_{47} values are reported on the carbon dioxide equilibrium scale[4] and normalised to an acid digestion temperature of 25°C from the original acid digestion temperature of 90 °C, using an acid fractionation factor of 0.081‰[5]. The difference between the [*Gonfiantini*] and the [*Brand*] $\delta^{13}C$ and $\delta^{18}O$ values are around 0.01‰ (see Supplementary Data 1). Standard errors (in brackets) for the isotope analyses are calculated on the 1σ level and for the trace element analyses on the 2σ level.

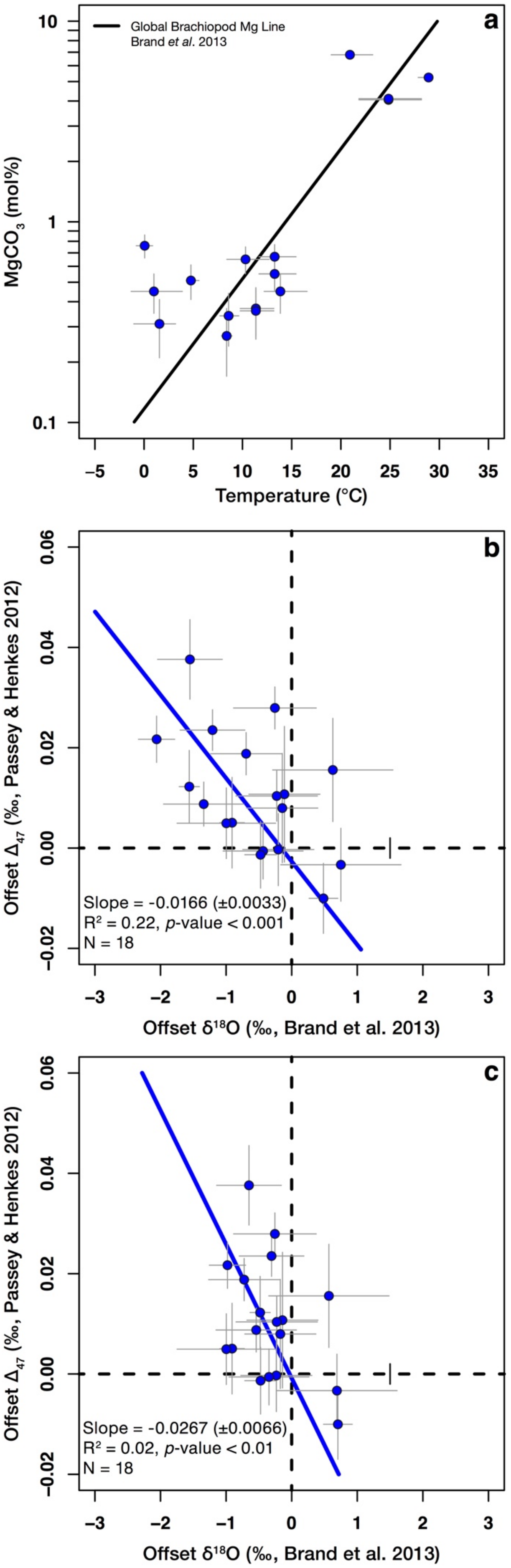

Supplementary Figure 1 | Effect of the magnesium concentration on the $\delta^{18}O$ values. (**a**) Magnesium concentrations of the brachiopod shells analysed in this study plotted against the corresponding brachiopod growth temperatures. Our results are consistent with the expected range of modern brachiopod calcite and fall along the Global Brachiopod Mg Line[3]. (**b,c**) Same as Figures 1b,c in the main article but the offset $\delta^{18}O$ values are calculated according to Brand *et al.*[3] that includes a correction for the Mg-effect, which accounts for a 0.17‰ change per mol% $MgCO_3$[6]. The range of the offset $\delta^{18}O$ values become larger, compared to those calculated according to Kim and O'Neil[7], if the Mg-effect is considered (Figs 1b,c in the main article). This suggests that the Mg-content of the brachiopod shells cannot account for the observed deviations from apparent oxygen isotope equilibrium. For all plots: linear regression lines fitted to our data consider the errors. Corresponding two-tailed *p*-values are computed using a t-test. Error bars for the offset $\delta^{18}O$ values indicate the mean deviation from oxygen isotope equilibrium calculated using the minimum and the maximum temperature estimates. Error bars for the offset Δ_{47} values indicate the 1σ S.E. of the replicate measurements.

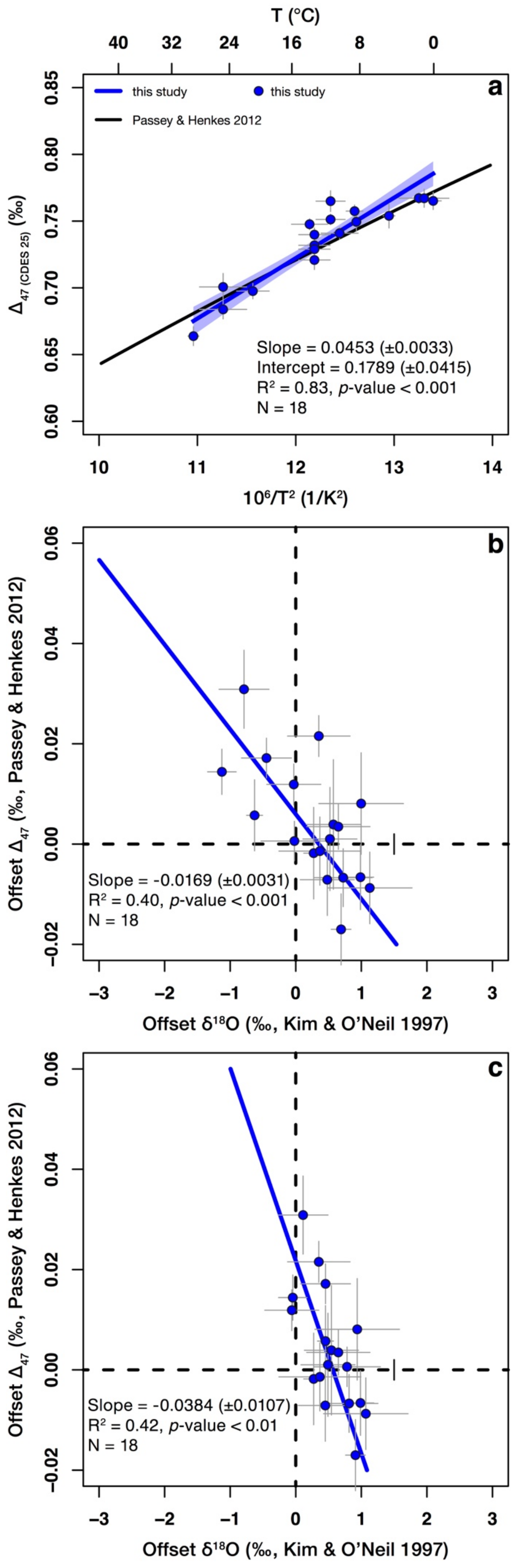

Supplementary Figure 2 | Brachiopods show an offset from equilibrium Δ_{47} and $\delta^{18}O$ values. Same as Figure 1 in the main article but all values were calculated using the [*Brand*] set of isotopic parameters[8]. (**a**) Δ_{47}–temperature dependence derived from the eighteen modern brachiopods analysed in this study. (**b**) The offset $\delta^{18}O$ and offset Δ_{47} values show a significant negative correlation. Seawater $\delta^{18}O$ values were acquired from the Global Seawater Oxygen-18 Database[2]. (**c**) The correlation between offset $\delta^{18}O$ and offset Δ_{47} values is still present if, where available, the directly measured seawater $\delta^{18}O$ values (Supplementary Table 1) were used for the calculations. For all plots: linear regression lines fitted to our data consider the errors. Corresponding two-tailed *p*-values are computed using a t-test. Error bars for the offset $\delta^{18}O$ values indicate the mean deviation from oxygen isotope equilibrium calculated using the minimum and the maximum temperature estimates (Supplementary Table 1). Error bars for the offset Δ_{47} values indicate the 1σ S.E. of the replicate measurements.

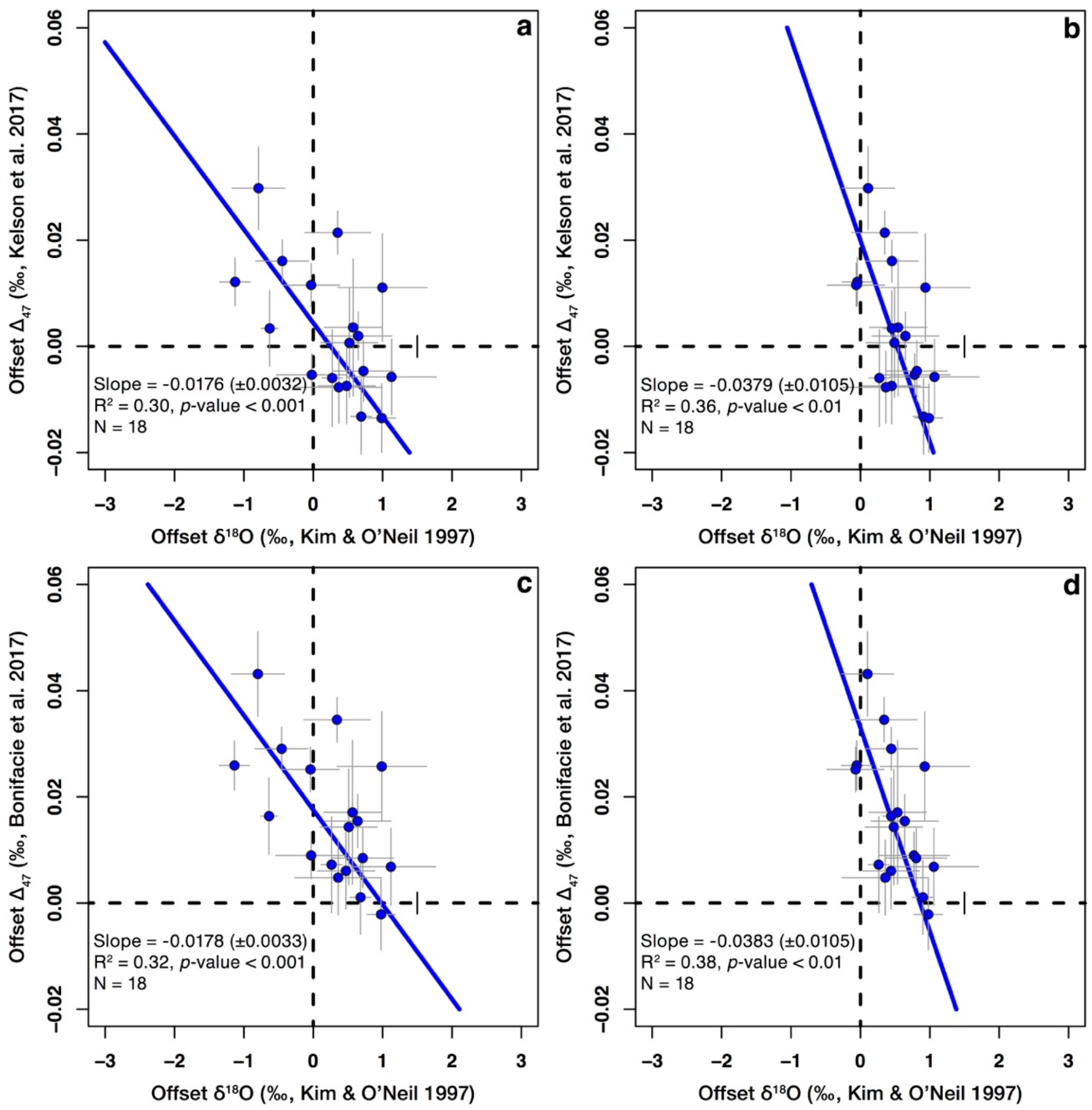

Supplementary Figure 3 | Brachiopods show an offset from equilibrium Δ_{47} and $\delta^{18}O$ values irrespective of the calibration characteristic of clumped isotope equilibrium. The offset $\delta^{18}O$ and the offset Δ_{47} values show a significant negative correlation even if clumped isotope equilibrium is assumed to be represented by the experimental calibrations of Bonifacie *et al.*[9], i.e., their eq. 3, or Kelson *et al.*[10], i.e., their eq. 1. The dataset of Kelson *et al.*[10] represents the first calibration where raw data was processed using the [*Brand*] set of isotopic parameters[8]. (**a,c**) All seawater $\delta^{18}O$ values were acquired from the Global Seawater Oxygen-18 Database[2]. (**b,d**) Where available, the directly measured seawater $\delta^{18}O$ values were used for the calculations[3]. For all plots: linear regression lines fitted to our data consider the errors. Corresponding two-tailed *p*-values are computed using a t-test. Error bars for the offset $\delta^{18}O$ values indicate the mean deviation from oxygen isotope equilibrium calculated using the minimum and the maximum temperature estimates. Error bars for the offset Δ_{47} values indicate the 1σ S.E. of the replicate measurements.

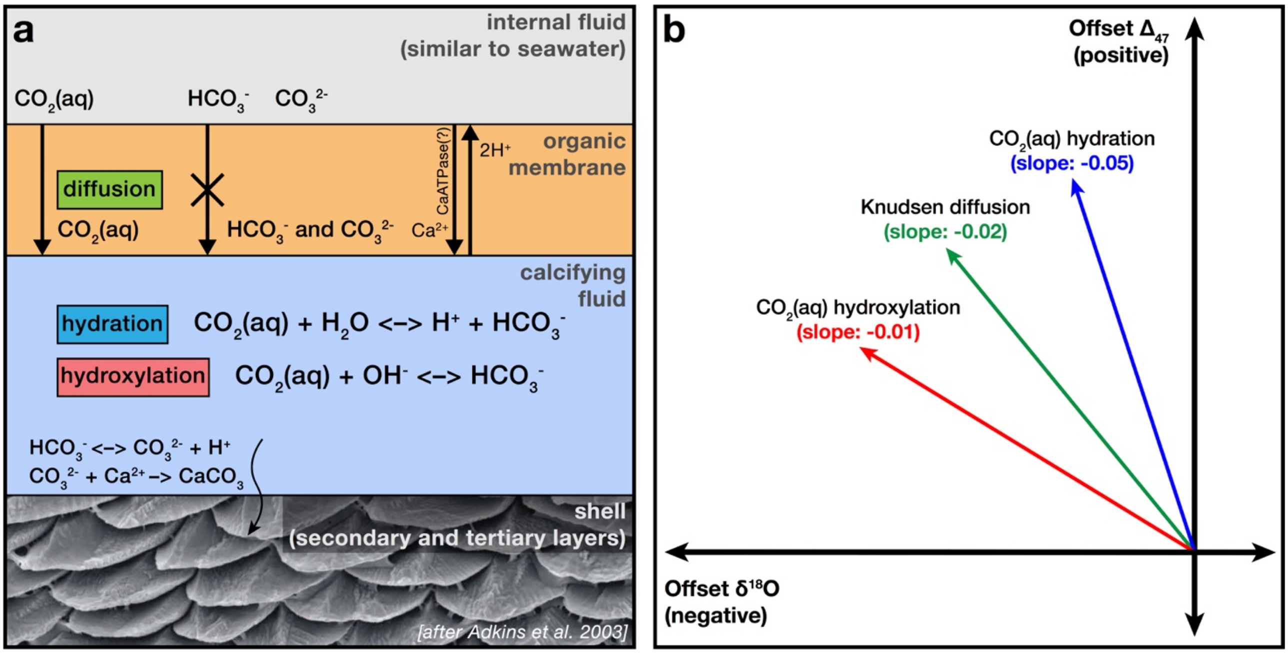

Supplementary Figure 4 | A model explaining the possible causes of kinetic effects occurring during biogenic calcite precipitation in modern brachiopods. (**a**) A simplified model of biogenic calcite precipitation in brachiopods[11,12]. Isotope fractionation occurs during the diffusion of $CO_{2(aq)}$ through an organic membrane and during the transformation of $CO_{2(aq)}$ to bicarbonate (HCO_3^-) via hydration and hydroxylation reactions. For a detailed discussion, see the main text. (**b**) Each specific kinetic effect causes a different gradient on the plot between offset $\delta^{18}O$ and offset Δ_{47}[13-16].

Clumped isotope analyses

Clumped isotope analyses were made between July 2016 and April 2017, using a fully automated gas extraction and purification line connected to a ThermoFisher MAT 253 gas-source isotope-ratio mass spectrometer at the Goethe University, Frankfurt, Germany. Each sample was analysed in 4–10 replicates. At the extraction line, 4–8 mg of homogenised carbonate powder was reacted for 30 minutes at 90 °C with > 105% phosphoric acid. The resultant CO_2 was lead through a U-trap submerged into ethanol, cooled to -80 °C and frozen out immediately using a second U-trap that was submerged into liquid nitrogen. On completion of the reaction, the U-trap containing the solid CO_2 was submerged into -80 °C ethanol and a third U-trap was submerged into liquid nitrogen. The CO_2 sublimes from the ethanol-cooled U-trap and freezes out in the subsequent liquid-nitrogen-cooled U-trap, while water stays frozen in the U-trap kept at -80°C. The process of CO_2 sublimation at -80 °C was repeated altogether three times. For further purification, the CO_2 gas was entrained into a helium carrier gas and led through a Porapak Q trap, cooled down to -15 °C, to filter out hydrocarbons and other contaminants. Finally, the helium carrier gas was pumped away and the CO_2 gas enters the dual inlet system of the mass spectrometer, where it was analysed alternately with a reference gas of known isotope composition (Alphagas Izotop, Air Liquide, Paris, France; $\delta^{18}O_{VSMOW}$ = 25.56‰, $\delta^{13}C_{VPDB}$ = -4.30‰). Each analysis output of the mass spectrometer consisted of 10 acquisitions, made up of 10 cycles with 20 s integration time each and an additional pre-measurement of the reference gas. In each cycle, the peak intensities were measured for m/z 44 through m/z 49 for both the sample and the reference gas. Bellow pressure was adjusted to 16,000(±150) mV for m/z 44 before each acquisition. Background correction was performed for the sample and the reference gas separately, as described in Fiebig *et al.*[17]. Isobaric contaminant masses, monitored by comparing the correlation of off-peak m/z 47 and on-peak m/z 49 intensities for both the sample and the reference gas, were not observed (Supplementary Fig. 4).

For all samples, at least 4 replicates have been measured. The corresponding shot noise limit for 4 replicates is 0.004‰ and further decreases with increasing number of replicates[18]. For each sample analysed the 1σ standard error is always larger than the corresponding shot noise limit. Raw Δ_{47} data was transferred to the absolute reference frame of Dennis *et al.*[4] using empirical transfer functions (ETF). Two ETFs were used during this study (Supp. Data 1):

Supplementary Table 3 | Empirical transfer functions used in this study

	Slope [*Gonfiantini*]	Intercept [*Gonfiantini*]	Slope [*Brand*]	Intercept [*Brand*]
06.06.2016 – 12.22.2016	1.0797	0.9627	1.0586	0.9432
01.06.2017 – 04.05.2017	1.0981	0.9720	1.0739	0.9496

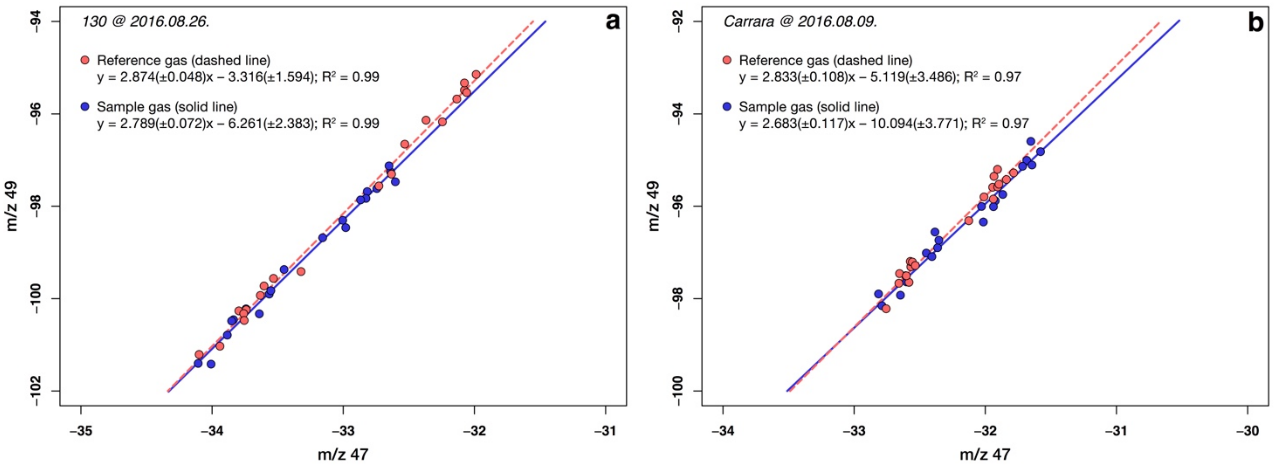

Supplementary Figure 5 | Background correction. (**a**) Correlation between intensity of the on-peak m/z 49 ion beam and the corresponding off-peak m/z 47 background for a replicate analysis of the sample that shows the highest Δ_{47} offset with respect to Passey and Henkes[5]. (**b**) Correlation between intensity of the on-peak m/z 49 ion beam and the corresponding off-peak m/z 47 background for a replicate analysis of a Carrara standard. If the sample gas would contain more m/z 49 interferences than the reference gas, the two regression lines would become distinguishable from each other. In this case, the sample gas regression line would shift to the right relative to the reference gas regression line[17,19]. Note that in both cases the slope and intercept of reference gas and sample gas measurements agree within errors. As such there are no indications for isobaric interferences on m/z 49.

Oxygen isotope analyses with ion probe (SIMS)

The ion probe analyses were carried out using the Caméca IMS 1280-HR2 at CRPG-CNRS (Nancy, France) and the method described in detail in Rollion-Bard *et al.*[20]. Oxygen isotope compositions were analysed using a 5 nA Cs^+ primary beam with a charge compensation by a normal-incidence electron gun and mass resolving power of M/ΔM ~5000. Oxygen isotopes were measured simultaneously in multi-collection mode by using two off-axis Faraday cups, L’2 and H1. Gains of Faraday cups were calibrated at the beginning of the analytical session. Each analysis was performed with a pre-sputtering time of 30 seconds followed by 30 cycles of data collection, 4 seconds each. Typical ion intensities of 6 x 10^6 cps and 3 x 10^9 cps were obtained on $^{18}O^-$ and $^{16}O^-$, respectively. After few minutes of counting, the internal $2\sigma_n$ error was less than ±0.1‰. An in-house carbonate standard (CCciAg; $\delta^{18}O_{VSMOW}$ = 18.94‰; $\delta^{13}C_{VPDB}$ = -11.61‰) was measured before and after each analytical session to correct for instrumental mass fractionation (IMF). The external reproducibility (1σ S.D.), based on the replicates of the carbonate standard was between ±0.27–0.40‰, depending on the analytical session. The IMF of sample was also corrected from Mg-content by applying the correction -0.3 x $MgO\%_{wt}$[21]. In addition to the ion probe analyses, the $\delta^{18}O$ values of the secondary layer of the studied shells were also determined by conventional mass spectrometry and an adjustment was applied to the ion probe $\delta^{18}O$ values as shown in Cusack *et al.*[22].

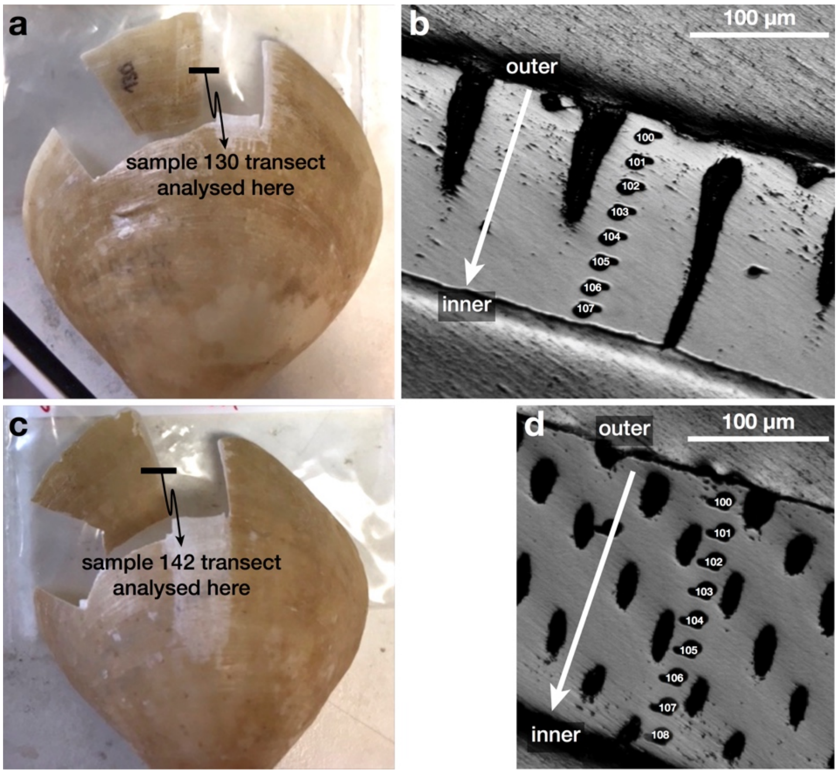

Supplementary Figure 6 | Location of the ion probe measurements on the two analysed *M. venosa* shells. (**a**,**b**) Sample 130. (**c**,**d**) Sample 142. In both cases, the ventral valves were analysed.

Oxygen isotope fractionation factors

Our $\delta^{18}O$ values for calcite were obtained applying a 90 °C acid fractionation factor of 1.00813, extrapolated from Kim *et al.*[23]. For 25 °C a fractionation factor of 1.01031 is obtained according to the same study. In their original calibration Kim and O'Neil[7] applied a 25 °C fractionation factor of 1.01050. To calculate the oxygen isotope fractionation between seawater and calcite we used the following modified equation of Kim and O'Neil[7]:

(S1) $$1000\ln\alpha_{cc-water} = 18.03 \times (1000/T) - 32.23,$$

where α is the fractionation factor and T is the temperature in K. Note that the original intercept of -32.42 has been corrected by +0.19, which takes into account the difference in the 25 °C acid fractionation factors of Kim *et al.*[23] relative to Kim and O'Neil[7].

References

1 Locarnini, R. A. *et al. World Ocean Atlas 2013, Volume 1: Temperature*. (2013).

2 LeGrande, A. N. & Schmidt, G. A. Global gridded data set of the oxygen isotopic composition in seawater. *Geophys. Res. Lett.* **33**, 1-5 (2006).

3 Brand, U. *et al.* Oxygen isotopes and $MgCO_3$ in brachiopod calcite and a new paleotemperature equation. *Chem. Geol.* **359**, 23-31 (2013).

4 Dennis, K. J., Affek, H. P., Passey, B. H., Schrag, D. P. & Eiler, J. M. Defining an absolute reference frame for 'clumped' isotope studies of CO_2. *Geochim. Cosmochim. Acta* **75**, 7117-7131 (2011).

5 Passey, B. H. & Henkes, G. A. Carbonate clumped isotope bond reordering and geospeedometry. *Earth Planet. Sci. Lett.* **351-352**, 223-236 (2012).

6 Jiménez-López, C., Romanek, C. S., Huertas, F. J., Ohmoto, H. & Caballero, E. Oxygen isotope fractionation in synthetic magnesian calcite. *Geochim. Cosmochim. Acta* **68**, 3367-3377 (2004).

7 Kim, S.-T. & O'Neil, J. R. Equilibrium and nonequilibrium oxygen isotope effects in synthetic carbonates. *Geochim. Cosmochim. Acta* **61**, 3461-3475 (1997).

8 Daëron, M., Blamart, D., Peral, M. & Affek, H. P. Absolute isotopic abundance ratios and the accuracy of Δ_{47} measurements. *Chem. Geol.* **442**, 83-96 (2016).

9 Bonifacie, M. *et al.* Calibration of the dolomite clumped isotope thermometer from 25 to 350 °C, and implications for a universal calibration for all (Ca, Mg, Fe)CO_3 carbonates. *Geochim. Cosmochim. Acta* **200**, 255-279 (2017).

10 Kelson, J. R., Huntington, K. W., Schauer, A. J., Saenger, C. & Lechler, A. R. Toward a universal carbonate clumped isotope calibration: Diverse synthesis and preparatory methods suggest a single temperature relationship. *Geochim. Cosmochim. Acta* **197**, 104-131 (2017).

11 Adkins, J. F., Boyle, E. A., Curry, W. B. & Lutringer, A. Stable isotopes in deep-sea corals and a new mechanism for "vital effects". *Geochim. Cosmochim. Acta* **67**, 1129-1143 (2003).

12 Simkiss, K. & Wilbur, K. M. *Biomineralization - Cell biology and mineral deposition*. (Academic Press, 1989).

13 Spooner, P. T. *et al.* Clumped isotope composition of cold-water corals: A role for vital effects? *Geochim. Cosmochim. Acta* **179**, 123-141 (2016).

14 Thiagarajan, N., Adkins, J. & Eiler, J. Carbonate clumped isotope thermometry of deep-sea corals and implications for vital effects. *Geochim. Cosmochim. Acta* **75**, 4416-4425 (2011).

15 Loyd, S. J. *et al.* Methane seep carbonates yield clumped isotope signatures out of equilibrium with formation temperatures. *Nat. Commun.* **7**, 1-12 (2016).

16 Guo, W., Kim, S., Thiagarajan, N., Adkins, J. F. & Eiler, J. M. in *American Geophysical Union Fall Meeting* PP34B-07 (2009).

17 Fiebig, J. *et al.* Slight pressure imbalances can affect accuracy and precision of dual inlet-based clumped isotope analysis. *Isot. Environ. Health Stud.* **52**, 12-28 (2016).

18 Merrit, D. A. & Hayes, J. M. Factors controlling precision and accuracy in isotope-ratio-monrtoring mass spectrometry. *Analytical Chemistry* **66**, 2336-2347 (1994).

19 Bernasconi, S. M. *et al.* Background effects on Faraday collectors in gas-source mass spectrometry and implications for clumped isotope measurements. *Rap. Commun. Mass Spec.* **27**, 603-612 (2013).

20 Rollion-Bard, C., Mangin, D. & Champenois, M. Development and application of oxygen and carbon isotopic measurements of biogenic carbonates by ion microprobe. *Geostand. Geoanal. Res.* **31**, 39-50 (2007).

21 Rollion-Bard, C. & Marin-Carbonne, J. Determination of SIMS matrix effects on oxygen isotopic compositions in carbonates. *J. Anal. At. Spectrom.* **26**, 1285-1289 (2011).

22 Cusack, M., Huerta, A. P. & EIMF. Brachiopods recording seawater temperature - A matter of class or maturation? *Chem. Geol.* **334**, 139-143 (2012).

23 Kim, S.-T., Mucci, A. & Taylor, B. E. Phosphoric acid fractionation factors for calcite and aragonite between 25 and 75 °C: Revisited. *Chem. Geol.* **246**, 135-146 (2007).
